# CRIS: complete reconstruction of immunoglobulin *V-D-J* sequences from RNA-seq data

**DOI:** 10.1093/bioadv/vbab021

**Published:** 2021-09-09

**Authors:** Rashedul Islam, Misha Bilenky, Andrew P Weng, Joseph M Connors, Martin Hirst

**Affiliations:** 1 Bioinformatics Graduate Program, University of British Columbia, Vancouver, BC V5Z 4S6, Canada; 2 Department of Microbiology and Immunology, Michael Smith Laboratories, University of British Columbia, Vancouver, BC V6T 1Z3, Canada; 3 Canada’s Michael Smith Genome Sciences Centre, BC Cancer, Vancouver, BC V5Z 4S6, Canada; 4 Terry Fox Laboratory, BC Cancer, Vancouver, BC V5Z 1L3, Canada; 5 Department of Pathology & Laboratory Medicine, University of British Columbia, Vancouver, BC V6T 2B5, Canada; 6 Department of Medical Oncology, BC Cancer, Vancouver, BC, V5Z 4E6, Canada

## Abstract

**Motivation:**

B cells display remarkable diversity in producing B-cell receptors through recombination of immunoglobulin (Ig) *V-D-J* genes. Somatic hypermutation (SHM) of immunoglobulin heavy chain variable (*IGHV*) genes are used as a prognostic marker in B-cell malignancies. Clinically, *IGHV* mutation status is determined by targeted Sanger sequencing which is a resource-intensive and low-throughput procedure. Here, we describe a bioinformatic pipeline, CRIS (Complete Reconstruction of Immunoglobulin *IGHV-D-J* Sequences) that uses RNA sequencing (RNA-seq) datasets to reconstruct *IGHV-D-J* sequences and determine *IGHV* SHM status.

**Results:**

CRIS extracts RNA-seq reads aligned to Ig gene loci, performs assembly of Ig transcripts and aligns the resulting contigs to reference Ig sequences to enumerate and classify SHMs in the *IGHV* gene sequence. CRIS improves on existing tools that infer the B-cell receptor repertoire from RNA-seq data using a portion *IGHV* gene segment by *de novo* assembly. We show that the SHM status identified by CRIS using the entire *IGHV* gene segment is highly concordant with clinical classification in three independent chronic lymphocytic leukemia patient cohorts.

**Availability and implementation:**

The CRIS pipeline is available under the MIT License from https://github.com/Rashedul/CRIS.

**Supplementary information:**

Supplementary data are available at *Bioinformatics Advances* online.

## 1 Introduction

**Fig. 1.**
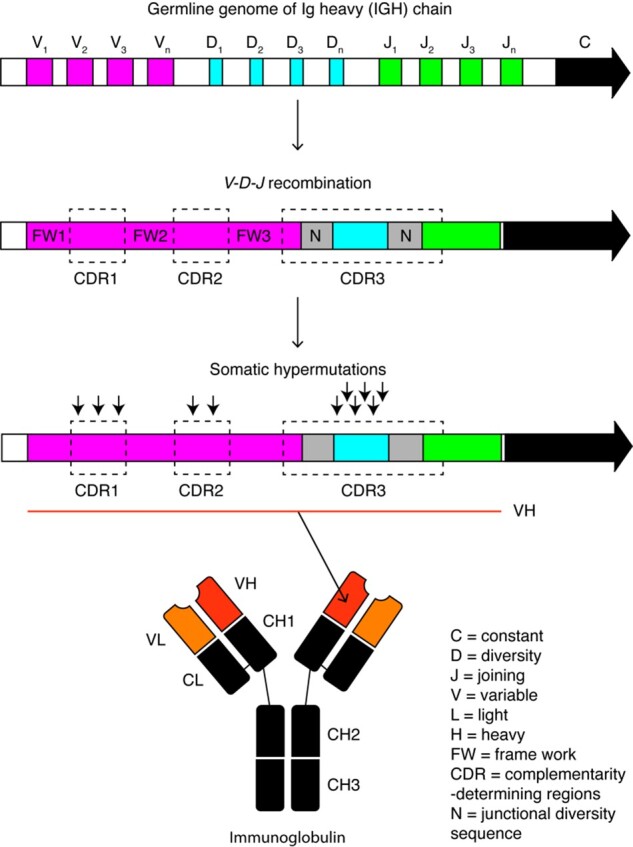
*IGHV-D-J* recombination and SHM during B-cell development. BCRs are generated by ordered assembly of the Ig heavy chain gene segments (*V*, *D* and *J*) during B-cell development. Addition and deletion of junctional nucleotides (N) contribute to the diversity of BCR repertoires. BCR sequences undergo affinity maturation upon antigen stimulation through SHMs in the variable domain (indicated in black arrows). SHMs of Ig are enriched at the complementarity-determining regions (CDRs)

During development in the bone marrow, B lymphocytes undergo rearrangement of immunoglobulin (Ig) heavy (V, D and J) and light chain (V and J) gene segments through recombination (Fig. 1). Addition or deletion of nucleotides occurs at segment junctions during recombination. In the germinal center, B-cells acquire additional somatic hypermutation (SHM) within the Ig variable regions as part of the adaptive immune response to generate a B-cell receptor (BCR) repertoire diversity estimated to be as much as ∼10^18^ (Briney *et al.*, 2019; Janeway *et al.*, 2004). Following SHM, B cells are positively selected for further differentiation into memory B cells or antibody-secreting plasma cells (Akkaya *et al.*, 2020).

Profiling of the B-cell Ig repertoire has become an essential component of immune research and is used clinically for malignant B-cell classification (Briney *et al.*, 2019; Georgiou *et al.*, 2014). B-cell malignancies arise at different stages of B-cell development and BCR diversification is used as both a prognostic and diagnostic marker (Georgiou *et al.*, 2014; Monk *et al.*, 2017). The presence of SHM and specific usage of immunoglobulin heavy chain variable (*IGHV*) genes are prognostic markers in different B-cell malignancies, including chronic lymphocytic leukemia (CLL), mantle cell lymphoma (MCL) and follicular lymphoma (Berget *et al.*, 2015; Damle *et al.*, 1999; Hamblin *et al.*, 1999; Navarro *et al.*, 2012). Malignant B cells are classified into two major subtypes based on the SHM status, where cells with very low SHM are classified as ‘unmutated *IGHV*’ subtype, while those cells with evidence of SHM are classified as ‘mutated *IGHV*’ subtype. Unmutated *IGHV* subtypes of CLL and MCL show more aggressive disease compared to the mutated *IGHV* subtype (Damle *et al.*, 1999; Hamblin *et al.*, 1999; Navarro *et al.*, 2012). *IGHV* gene usage is also used as a prognostic in follicular lymphoma (Berget *et al.*, 2015).

SHM analysis of the *IGHV* gene is commonly performed using multiplex PCR and Sanger sequencing following the best practice guidelines by the European Research Initiative on CLL (ERIC) (Ghia *et al.*, 2007). However the PCR-Sanger method is resource-intensive and technically challenging in both clinical and research applications and suffers from a 9% to 18% failure rate (Stamatopoulos *et al.*, 2017). Massively parallel sequencing of targeted genomic DNA regions or RNA has emerged as an alternative method to reliably sequence *V-D-J* segments (Boyd and Joshi, 2014; Georgiou *et al.*, 2014; Menzel *et al.*, 2014; Yaari and Kleinstein, 2015). RNA sequencing (RNA-seq) has become the gold standard for transcriptome analysis, applied in both clinical and research settings and has been used in limited cases to identify BCR rearrangement repertoire (Blachly *et al.*, 2015; Iglesia *et al.*, 2014; Monk *et al.*, 2017; Mose *et al.*, 2016).

Several bioinformatic pipelines have been developed to infer BCR repertoire from RNA-seq data, including ABRA (Iglesia *et al.*, 2014), TRUST (Hu *et al.*, 2019), ImReP (Mandric *et al.*, 2020), MiXCR (Bolotin *et al.*, 2015), V’DJer (Mose *et al.*, 2016) and IgID (Blachly *et al.*, 2015). Among them, ABRA (Iglesia *et al.*, 2014) and IgID (Blachly *et al.*, 2015) were not published with stand-alone code to allow for replication. The remaining *IGHV-D-J* reconstruction tools (e.g. TRUST, ImReP, MiXCR and V'DJer) were designed to reconstruct only the CDR3 region, representing only a portion of the *IGHV* gene, while the entire *IGHV* gene segment is required to determine the SHM status in B-cell malignancies. In addition, these tools have not been validated against gold standard PCR-Sanger datasets for SHM classification. To address these gaps in determining *IGHV* mutational status in B-cell malignancies, we developed a bioinformatic pipeline, CRIS (Complete Reconstruction of Immunoglobulin *IGHV-D-J* Sequences), which extracts RNA-seq reads aligned to putative Ig loci, assembles the complete *IGHV* gene, identifies the most abundant Ig transcript and enumerates SHMs by comparison with germline reference sequences. Classification of *IGHV* mutational subtypes by CRIS was validated against PCR-Sanger-based clinical classification in three independent cohorts of CLL patients and shown to be comparable.

## 2 Methods

### 2.1 CLL samples

In the Centre for Epigenomic Technology (CEMT) cohort, peripheral blood samples were obtained from CLL patients undergoing treatment at BC Cancer (*n* = 16) and used according to procedures approved by the Research Ethics Board (REB H12-01767) of the University of British Columbia (Supplementary Table S1). RNA was purified from those peripheral blood samples and extraction was performed on CD19+ sorted cells with >90% purity as described (Pellacani *et al.*, 2016).

### 2.2 RNA sequencing

The CEMT CLL RNA-seq datasets were generated as described (Pellacani *et al.*, 2016). RNA extraction, library construction and sequencing were performed following the guidelines formulated by the International Human Epigenome Consortium (http://www.ihec-epigenomes.org). These guidelines as well as the standard operating procedures for RNA-seq library construction and sequencing are available at https://thisisepigenetics.ca/for-scientists/protocols-and-standards and by request. Additional CLL patient RNA-seq datasets with matching *IGHV* mutation status were collected from published datasets: GSE66228 (Blachly *et al.*, 2015), EGAD00001004046 (Beekman *et al.*, 2018) and phs000435.v3 (Wang *et al.*, 2011).

### 2.3 Identification of putative Ig loci

We identified five putative Ig loci enriched with reads that were used to reconstruct Ig containing contigs in the 16 CEMT samples (Table 1). The detailed procedure of identifying Ig loci is described in Supplementary Figure S1a.

**Table 1.** Genomic coordinates of the putative Ig loci in the GRCh38 reference

Chromosome/contig	Start	End	Length (bp)
Chr14	105 550 001	106 880 000	1 329 999
Chr15	21 710 000	22 190 000	480 000
Chr16	31 950 001	33 970 000	2 019 999
chr14_KI270726v1_random	1	43 739	43 739
chr16_KI270728v1_random	1	1 872 759	1 872 759

### 2.4 CRIS pipeline

**Fig. 2.**
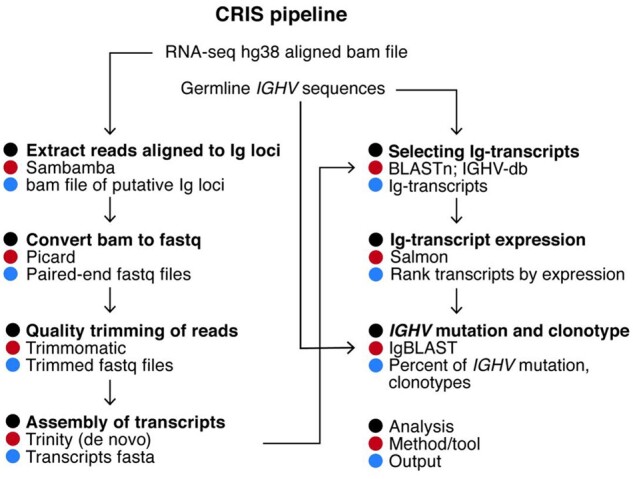
CRIS workflow. CRIS extract reads from the putative Ig loci prior to assembly of Ig transcripts and quantify transcript abundances. The percent of *IGHV* mutations of Ig transcripts is calculated by comparing to the germline sequences

- **Step 1:** *Read extraction prior to assembly of Ig* *transcripts:* hg38-bam-file was created by aligning the reads to the GRCh38 reference genome using BWA mem (v0.7.6a; Li and Durbin, 2009). Using sambamba (v0.7.0; Tarasov *et al.*, 2015), we extracted reads that were aligned to the putative *IGHV* loci (**Table 1**) and saved them in fastq format using Picard SamToFastq (v2.20.3; Broad Institute, 2009; **Fig. 2**). These resultant paired-end reads originated from the putative Ig loci were used as input for Trinity (v2.1.1; Grabherr *et al.*, 2011) for *de novo* transcriptome assembly.
- **Step 2:** *Identification of Ig* *transcripts and their abundances:* Trinity assembly performed in the previous step produced around 250 transcripts per sample. To filter the transcripts that have similarity (expectation value ≤20) with the germline *IGHV* sequences, we used blastn (v2.9.0; Altschul *et al.*, 1990) with default parameters with a custom database of *IGHV* sequences downloaded from the international ImMunoGeneTics information system (IMGT) (Giudicelli *et al.*, 2005). The resultant Ig transcripts were used in Salmon (v0.8.1; Patro *et al.*, 2017) to quantify their abundances with a k-mer of 31 bp. Transcript with the highest TPM (transcripts per million) value was marked as the dominant clone.
- **Step 3:** *SHM and clonotype analysis:* The Ig-transcript sequences identified in step 2 were queried in IgBLAST (v1.14.0; Ye *et al.*, 2013) against the germline *V*, *D and J* gene database of IMGT. IgBLAST returned the percent identity of the *IGHV* segment of Ig transcripts compared to the germline alleles and clustered the similar Ig transcripts into clonotypes. Productive Ig transcript with highest TPM value was used to determine *IGHV* mutation status of CLL sample and further compared with available clinical PCR-Sanger data. Transcripts having TPM values within one log10 of the highest expressed transcript were also considered while comparing with the PCR-Sanger data according to (Blachly *et al.* 2015).

### 2.5 Analysis of SHM status using V’DJer, TRUST and MiXCR

V’DJer, TRUST (v3.0.3) and MiXCR (v3.0.3) were run on the RNA-seq bam file generated by STAR (v2.7.5a) aligner (Dobin *et al.*, 2013) using GRCh38 genome as reference. During STAR alignment ‘–outSAMunmapped Within’ was used to include the unmapped reads in the bam file. All three tools were run with default parameters to generate VDJ contigs of IGH. VDJ contigs were analyzed by IgBLAST to generate the percent identity of *IGHV* sequences compared to the germline database.

## 3 Results

### 3.1 *De novo* assembly-based Ig detection from RNA-seq

*De novo* assembly using Trinity (Grabherr *et al.*, 2011) for 16 deeply sequenced (∼300 M read pairs) CLL RNA-seq libraries generated an average of ∼450 000 contigs per sample with 6–29 contigs demonstrating *IGHV* sequence homology. However, *de novo* assembly of the complete RNA-seq read sets required significant computational resources (Hölzer and Marz, 2019) and thus we sought to identify the fraction of reads in the RNA-seq libraries corresponding to the Ig loci. Using the resulting assemblies, we found that on average 99.85% of the sequence reads used to reconstruct *IGHV* containing contigs originated from five putative Ig loci in the GRCh38 reference (Supplementary Table S2). These putative Ig loci consist of human Ig locus, Ig pseudogene loci and unlocalized contigs at chromosomes 14, 15 and 16 (Table 1 and Supplementary Fig. S2). This suggests that sequence reads used to reconstruct Ig sequence not only map to the reference Ig locus but also to pseudogene regions both within the current assembly and in unlocalized contigs. We hypothesized that this novel set of loci could be used as a highly specific filter to reconstruct *IGHV-D-J* sequence.

### 3.2 CRIS pipeline development

**Fig. 3.**
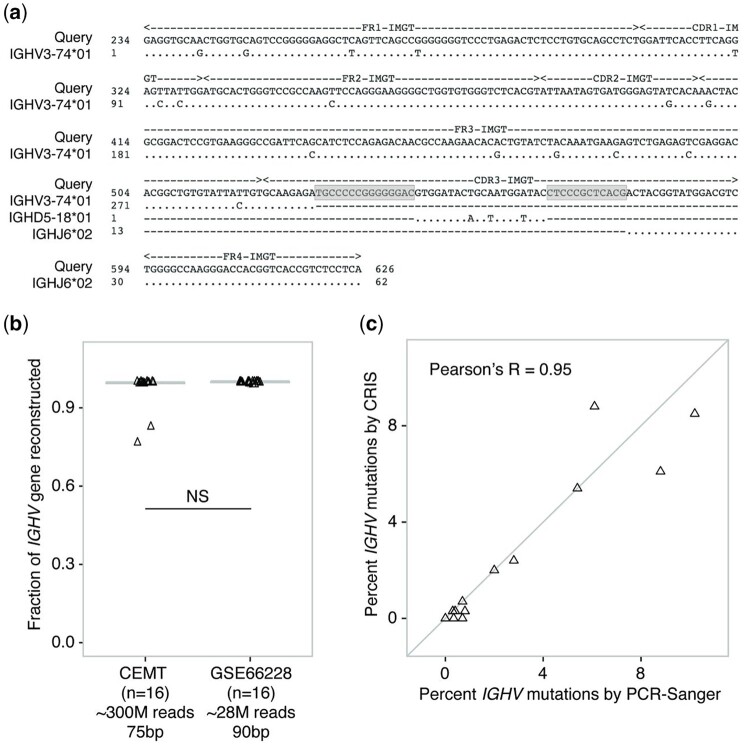
Evaluation of CRIS to reconstruct *IGHV-D-J* sequences. (**a**) The most abundant Ig transcript from US-1422278 sample was aligned to the germline database using IgBLAST where top hit germline genes are shown. In the alignment, mismatches are represented as nucleotide bases and matches as dots. The alignment length, number of matches and mismatches are 296, 280 and 16, respectively. Total number of matched nucleotides between query and germline *IGHV* sequence is used to calculate percent identity e.g., 100*(280/296) = 96.4%. N-junctional sequences are highlighted in gray boxes. (**b**) Fraction of the *IGHV* gene assembled in two CLL RNA-seq datasets with different sequence depths and lengths as indicated. An unpaired two-tailed *t*-test demonstrated no significant (*P =* 0.15) difference between the two distributions (NS). (**c**) Scatter plot comparing the percent of mutation of *IGHV* as predicted by CRIS and clinical PCR-Sanger-based analysis for 16 CLL patient samples obtained from GSE66228

Given the time required to complete a full assembly from an RNA-seq library, we sought to extract Ig sequences prior to performing assembly. For this, we leveraged the putative Ig loci identified in our pilot set of libraries, retrieved sequences aligned by BWA mem (Li and Durbin, 2009) within these coordinates (∼1% of all reads) and subjected these to *de novo* assembly using Trinity (Grabherr *et al.*, 2011). Enriching for Ig sequences from the bulk RNA-seq sequence set reduced the run time for assembly by two orders of magnitude while not significantly impacting the subsequent SHM analysis of Ig transcripts (Supplementary Table S3). We confirmed that our approach also successfully assembled the *IGHV-D-J* and N-junctional segments (Fig. 3a, Table 2 and Supplementary Table S4).

**Table 2.** Concordance of *IGHV* gene prediction and percent mutation between PCR-Sanger-based analysis and CRIS

Sample ID	Sanger		CRIS					
	IGHV	Mutation (%)	IGHV	*IGHV* mutation (%)	IGHD	IGHJ	No. of Ig transcript	No. of clonotype
US-1422282	V1-69	0.4	IGHV1-69*04	0.3	IGHD6-19*01	IGHJ4*02	7	4
US-1422366	V1-18	0.34	IGHV1-18*04	0	IGHD3-3*01	IGHJ6*02	21	5
US-1422311	V3-11	2	IGHV3-11*01	2	IGHD4-17*01	IGHJ4*02	5	4
US-1422278	V3-74	5.4	IGHV3-74*01	5.4	IGHD5-18*01	IGHJ6*02	5	3
US-1422335	V4-59	10.2	IGHV4-59*02	8.5	IGHD3-10*01	IGHJ4*02	3	2
US-1422321	V3-66	0.7	IGHV3-66*02	0.7	NA	IGHJ4*02	9	4
US-1422333	V4-34	0	IGHV4-34*01	0	IGHD3-3*01	IGHJ6*02	6	3
US-1422356	V2-70	0.8	IGHV2-70*01	0.3	IGHD3-16*01	IGHJ3*02	15	8
US-1422368	V3-74	6.1	IGHV3-74*03	8.8	IGHD1-1*01	IGHJ5*02	2	2
US-1422309	V3-53	8.8	IGHV3-53*01	6.1	IGHD3-10*01	IGHJ6*03	4	3
US-1422302	V2-70	0.3	IGHV2-70*01	0.3	IGHD2-15*01	IGHJ4*02	20	4
US-1422351	V1-46	0	IGHV1-46*01	0	IGHD3-10*01	IGHJ4*02	6	3
US-1422314	V1-3	0.7	IGHV1-3*01	0	IGHD6-19*01	IGHJ4*02	5	3
US-1422342	V3-21	0	IGHV3-21*01	0	IGHD3-16*01	IGHJ4*02	4	2
US-1422350	V3-48	2.8	IGHV3-48*03	2.4	IGHD3-22*01	IGHJ4*02	3	2
US-1422352	V1-46	0	IGHV1-46*01	0	IGHD3-22*01	IGHJ6*02	17	4

*Notes*: CRIS reconstructed *V-D-J* segments of Ig transcripts and identified multiple transcripts per sample that belong to different clonotypes. NA is used in cases where *IGHD* genes were absent.

Having established that enrichment of sequences using our Ig feature set significantly reduced compute resources without a reduction in the sensitivity, we next examined the impact of RNA-seq sequencing depth. For this, we leveraged a set of 16 CLL RNA-seq libraries with an average of ∼28 million paired reads from GSE66228 (Blachly *et al.*, 2015) and compared these to the results obtained from our deeply sequenced CEMT libraries (∼300 million paired reads). We found no significant difference in the fraction of the *IGHV* gene assembled between deep and shallow RNA-seq libraries (Fig. 3b). This appears to be in part due to the high expression level of the dominating clone in the GSE66228 dataset (Blachly *et al.*, 2015) driving sufficient sequence read coverage (at least 10^4^ reads) for Trinity to assemble the Ig transcript. However, as expected, the overall number of Ig transcripts identified correlated with the sequencing depth. Based on this analysis, we developed a pipeline called CRIS and benchmarked its ability to call *IGHV* mutation status. In the CRIS pipeline, we automate the process of read extraction from our novel putative Ig coordinates, perform quality trimming of selected sequence reads, assemble transcripts, enumerate transcript abundances and identify somatic mutations using reference germline sequences for SHM classification.

### 3.3 CRIS is concordant with clinical *IGHV* mutation status

Having established that CRIS could efficiently assembly *IGHV* transcripts, we explored its ability to call SHM mutation status in CLL. Unmutated CLL (uCLL) is clinically defined by *IGHV* sequence alignments of >98% identity to the reference sequence (Georgiou *et al.*, 2014; Monk *et al.*, 2017). To benchmark CRIS against gold standard Sanger-based clinical classification, we analyzed a series of published RNA-seq libraries from CLL patients with matched Sanger sequencing classifications. CRIS reported SHM on clonally amplified Ig transcripts and its classification of mutated/unmutated CLL (mCLL/uCLL) showed perfect concordance with Sanger-based clinical calls in the GSE66228 dataset (Blachly *et al.*, 2015; Table 2). The percent mutations reported by CRIS and the clinical test were also highly correlated (Pearson’s *r* = 0.95, 95% CI 0.86–0.98; Fig. 3c). The reported *IGHV* mutational frequency was identical in 8/16 cases with the remaining cases showing small deviations (mean deviation 0.22%) that did not change the SHM classification. In seven of the eight divergent cases, the percent *IGHV* identity reported by the Sanger-based test was higher compared to CRIS (Table 2). Closer inspection of the alignments revealed that this likely an artifact in the Sanger calls due to incomplete *IGHV* coverage by the PCR product used as denominator to calculate percent identity (Blachly *et al.*, 2015). In addition to calling mCLL/uCLL status, CRIS also reported 2–8 dominant clonotypes in the GSE66228 dataset, a feature not detected by clinical Sanger-based classifiers.

**Fig. 4.**
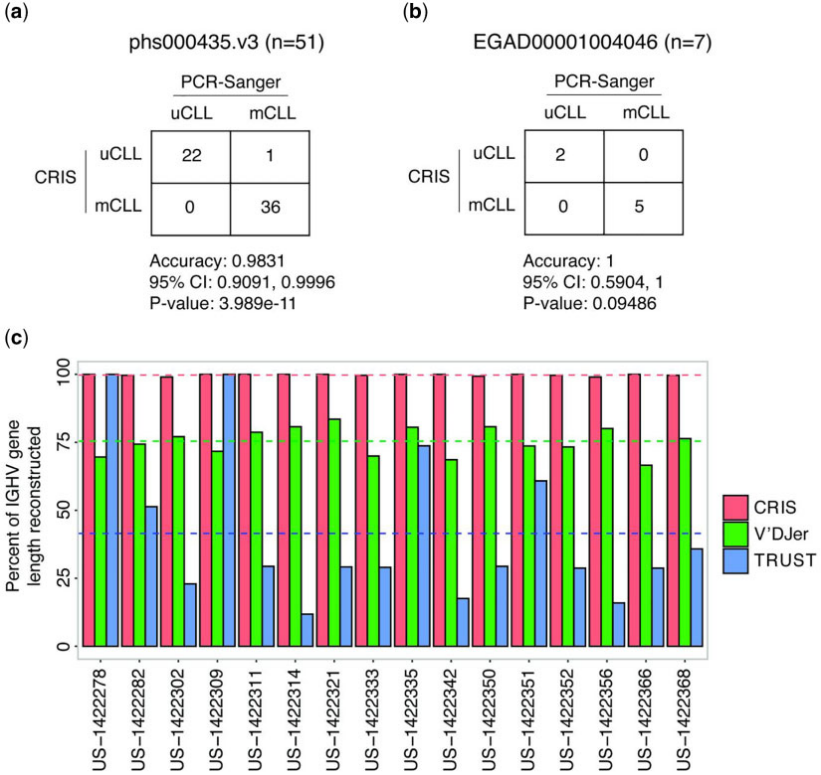
Comparison of CRIS with clinical data and existing tools. (**a and b**) Confusion matrix represents the classification accuracy of CRIS compared to Sanger-PCR data in two independent CLL cohorts. The *P*-value was calculated by one-sided binomial test. (**c**) Comparison of CRIS, V’DJer and TRUST to reconstruct the proportion of *IGHV* sequences in GSE66228 (Blachly *et al.*, 2015) dataset. The average fraction of IGHV gene length for each tool is represented by dashed horizontal lines

We further benchmarked CRIS using two independent CLL RNA-seq datasets with matched *IGHV* mutation status determined by Sanger sequencing. In the phs000435.v3 dataset (Wang *et al.*, 2011), CRIS calls were identical to the Sanger-based calls in 50/51 cases with 98.3% accuracy, 100% sensitivity and 97.3% specificity (Fig. 4a). A single sample (DFCI-5121) was reported as mCLL (Wang *et al.*, 2011), however, CRIS determined it as uCLL. In the third independent dataset, EGAD00001004046 (Beekman *et al.*, 2018), CRIS agreed with clinical classification in all cases and determined the identical *IGHV* gene as the dominant clone (Fig. 4b and Supplementary Fig. S1b).

### 3.4 Comparison of CRIS against existing tools

We next compared CRIS with previously published tools: V’DJer (Mose *et al.*2016), TRUST (Hu *et al.*, 2019) and MiXCR (Bolotin *et al.*, 2015) that reconstruct BCR repertoires from short-read RNA-seq data. In 16 CLL RNA-seq samples obtained from GSE66228 (Blachly *et al.*, 2015), V’DJer did not produce full-length *IGHV* as it is designed to generate contigs of fixed length (360 bp) spanning the CDR3 region. Thus, on average, V’DJer assembled 75.44% of the *IGHV* gene whereas CRIS reconstructed 99.74% (Fig. 4c). Partial reconstruction of the *IGHV* gene could lead to misclassification of *IGHV* mutation status especially for samples with *IGHV* sequence identity near the established 98% cutoff. For example, CRIS reconstructed 295 bp out of 296 bp of the IGHV3-74*03 sequence whereas V’DJer assembled 226 bp in US-1422368 (Supplementary Fig. S3a and b). The additional 69 bp reported by CRIS contained two mutations that resulted in a 1.2% difference in reported percent identity between CRIS (91.2%) and V’DJer (89.4%). TRUST assembled only 41.5% of the *IGHV* gene on average using the GSE66228 dataset (Fig. 4c). Furthermore, V’DJer and TRUST did not produce a contig for US-1422282 that contained *IGHV1-69* gene whereas CRIS generated *IGHV1-69* containing contig in agreement with the clinical call.

To compare the computational performance between CRIS and V’DJer, both of the pipelines were configured to use up to 16 threads. In the shallow libraries from GSE66228 dataset, CRIS had ∼14% faster total run time (average 3.07 wall-clock minutes) compared to V’DJer (average 3.50 wall-clock minutes). Using deeper RNA-seq datasets (∼300 million reads) V’DJer took five times more time to run than CRIS (87 versus 16 wall-clock minutes on average). Using 16 threads, TRUST took 36 wall-clock minutes on average using GSE66228, an order of magnitude longer than CRIS. MiXCR (Bolotin *et al.*, 2015) generated partial CDR3 sequence contigs with <10% of *IGHV* gene sequence in the GSE66228 dataset of 75 bp read length. MiXCR recommends ≥100 bp read length to extract CDR3 repertoires from RNA-Seq data. Thus, our comparisons suggest that existing BCR reconstruction tools developed to extract just CDR3 regions perform poorly compared to CRIS in the determination of SHM status because they are designed to generate and analyze partial *IGHV* sequences. Overall, CRIS showed increased sensitivity and specificity and reduced run time over existing RNA-seq-based BCR reconstruction tools.

## 4 Discussion

PCR-Sanger-based Ig SHM classification is resource-intensive, subject to PCR bias, and suffers from an ∼9% to 18% failure rate (Ghia *et al.*, 2007; Stamatopoulos *et al.*, 2017). In contrast, RNA-seq is now routinely applied in the clinical setting, eliminates the need for targeted amplification of Ig locus and can be used to identify BCR rearrangement repertoire (Blachly *et al.*, 2015). Here, we showed that CRIS can rapidly analyze RNA-seq to detect *IGHV* mutation status in CLL at a sensitivity and specificity equivalent to current Sanger-based clinical tests. Furthermore, CRIS was able to reconstruct the entire *IGHV* sequence thus increasing the accuracy of SHM classification. This is in contrast to a majority of existing pipelines designed to infer only CDR3-derived sequences (Bolotin *et al.*, 2015; Hu *et al.*, 2019; Mose *et al.*2016).

A registry of ∼1500 CLL patients showed that 90% of patients were not screened for *IGHV* mutations (Mato *et al.*, 2016). In the public domain, there are thousands of RNA-seq data available for different B-cell malignancies but their SHM status of *IGHV* genes is either not reported or partially reported. Furthermore, for a majority of publicly available RNA-seq datasets where SHM status is reported, detailed *IGHV* mutation reports with gene name, percent identity and clonal frequency are not available restricting the ability to assess mutational values. To meet this need, we developed CRIS and demonstrated its ability to rapidly classify *IGVH* mutational status with clinical accuracy. We anticipate that CRIS will prove to be useful in the mining of available B-cell RNA-seq datasets and that it will provide a framework to incorporate RNA-seq as a diagnostic tool to examine the BCR clonal rearrangement and SHM status.

## Supplementary Material

vbab021_Supplementary_Data
