# Supplementary material for: CRIS: complete reconstruction of immunoglobulin V-D-J sequences from RNA-seq data
Source: Bioinform Adv. 2021 Sep 9;1(1):vbab021. doi: 10.1093/bioadv/vbab021 (PMC8600631; doi:10.1093/bioadv/vbab021)
Supplement: vbab021_Supplementary_Data [file vbab021_supplementary_data.docx]

**CRIS:** **C**omplete **R**econstruction of **I**mmunoglobulin *V-D-J* **S**equences from RNA-seq data

**SUPPLEMENTARY**


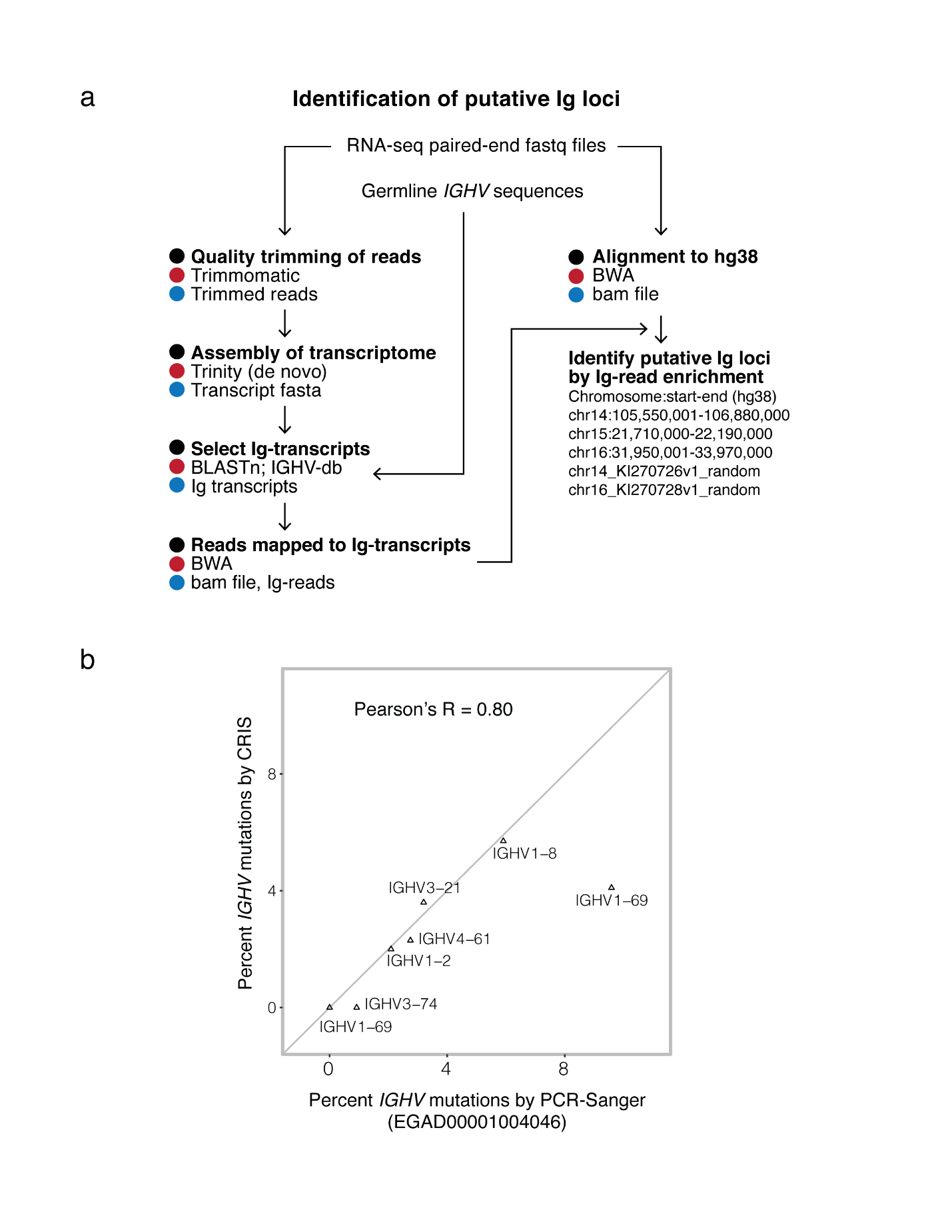


**Figure S1: Identification of putative Ig loci in the human reference genome.**

**a)** Reads were trimmed for low quality bases and adapters by Trimmomatic (Bolger *et al.*, 2014) before assembly. Trinity (v2.1.1) (Grabherr *et al.*, 2011) was run with the k-mer size of 25bp for whole transcriptome assembly of 16 CEMT RNA-seq samples. Contigs below the length of 200bp were discarded. From the whole transcriptome, transcripts that show similarity with the *IGHV* sequences were filtered using blastn (Altschul *et al.*, 1990) with default parameters. We used a custom database of *IGHV* sequences downloaded from IMGT (Ronique Giudicelli *et al.*). Ig-transcripts of each CLL sample were used as reference sequence to align RNA-seq reads of the corresponding sample using BWA mem (v0.7.6a) (Li and Durbin, 2009). The reads (Ig-reads) mapped to the Ig-transcripts were searched to the hg38-bam-file to find their genomic coordinates. Hg38-bam-file was generated by aligning paired-end RNA-seq reads to the human reference genome GRCh38 using BWA mem (v0.7.6a) (Li and Durbin, 2009). Ranking of chromosomes by the enrichment of Ig-reads identified five chromosomes/contigs e.g., chr14, chr15, chr16, chr14_KI270726v1_random and chr16_KI270728v1_random. We binned the chromosomes into 10Kb windows and counted the number of reads mapped to the bins for each sample. For each bin we took the average read counts among 16 CLL samples. We removed the bins with less than 20 mapped reads. Then we merged the neighboring bins within 1,000,000bp using bedtools (v2.29.0) (Quinlan and Hall, 2010). After merging the neighboring bins, we identified five genomic co-ordinates of putative Ig loci and those five regions were used to extract reads prior to de novo assembly of Ig-transcripts in the CRIS pipeline. **b)** Scatter plot comparing the percent of mutation of *IGHV* as predicted by CRIS and clinical PCR-Sanger based analysis for 7 CLL patient samples obtained from EGAD00001004046 (Beekman *et al.*, 2018).

**
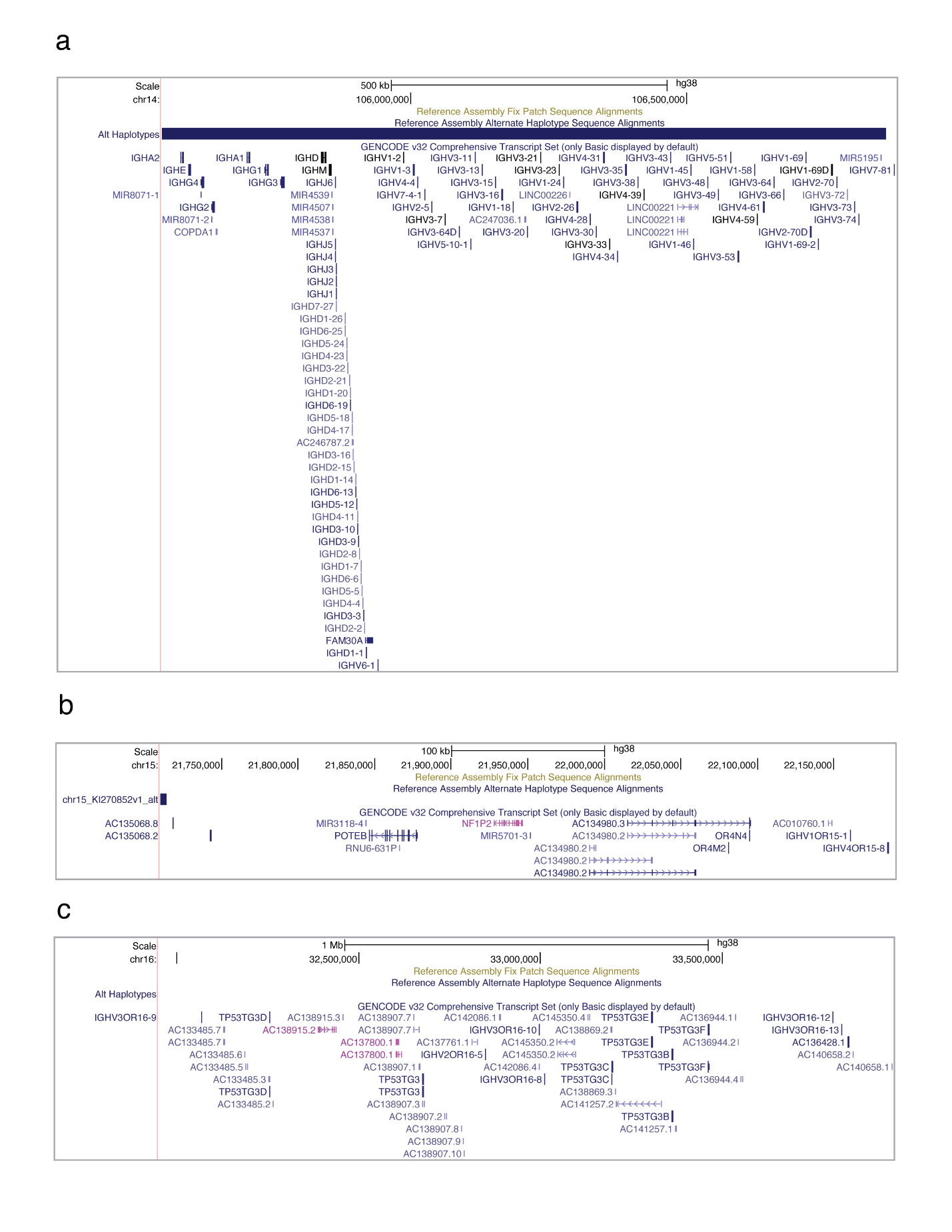
**

**Figure S2: Genes at putative Ig loci. a)** Genome browser screenshot for the genes in the human Ig locus at chromosome 14. **b,c)** Chromosome 15 and 16 have non-functional immunoglobulin heavy chain variable sequences e.g., IGHV1OR15-1, IGHV4OR15-8, IGHV1OR15-3 on chromosome 15 and IGHV3OR16-9, IGHV2OR16-5, IGHV3OR16-8, IGHV3OR16-10, IGHV3OR16-12, IGHV3OR16-13 on chromosome 16.

**
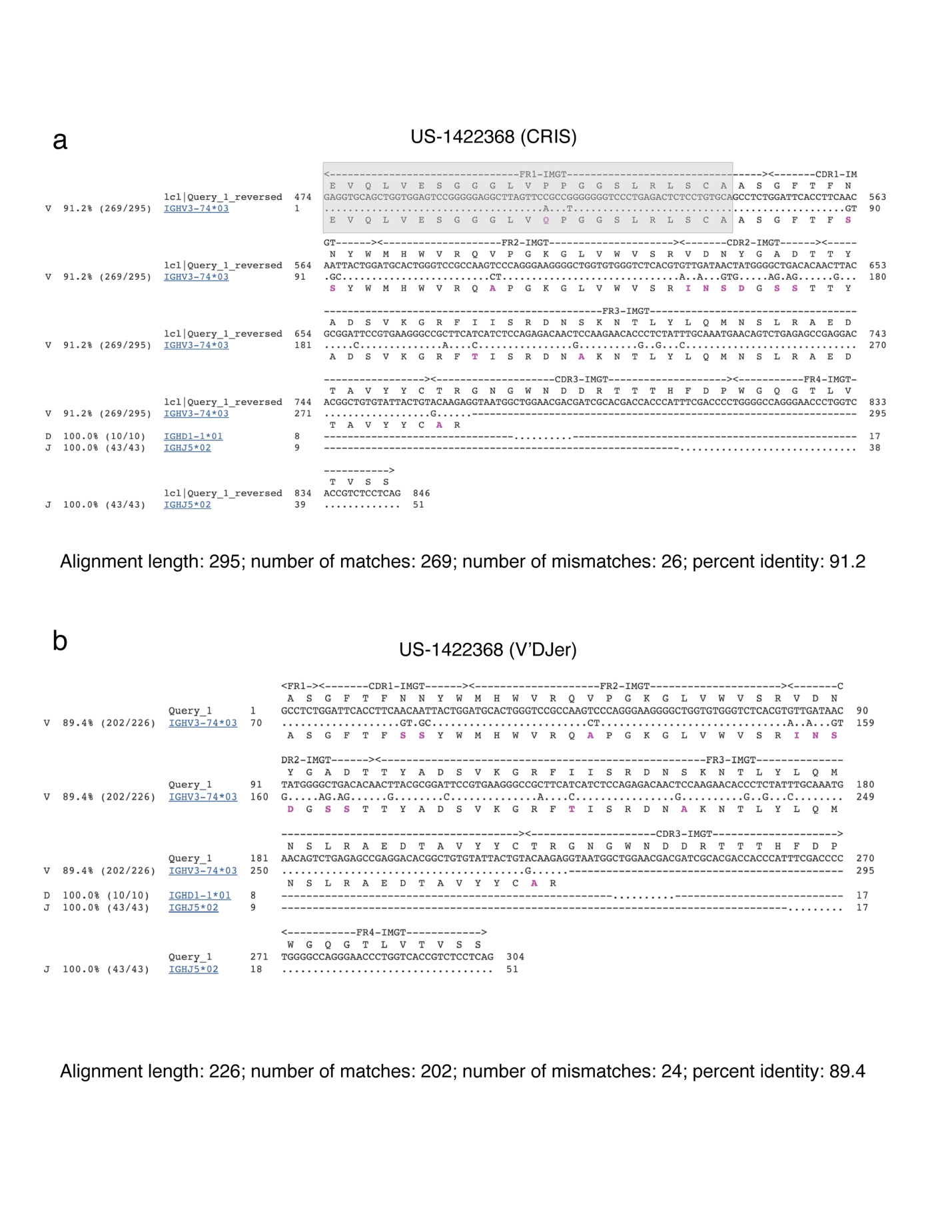
**

**Figure S3: Comparison between CRIS and V’DJer to reconstruct *IGHV* sequence. a,b)** CRIS reconstructed additional 69bp (highlighted in gray color) of *IGHV3-74*3* compared to V’DJer which changes the alignment length, total mutation counts and consequently the percent identity to the germline sequence.

**Table S1: CLL patient information.**

| **Study SubCode** | **AGE** | **SEX** | **DIAGNOSIS** |
| --- | --- | --- | --- |
| CEMT_1 | 70 | M | SLL |
| CEMT_25 | 62 | F | CLL |
| CEMT_26 | 72 | M | CLL |
| CEMT_27 | 65 | M | CLL |
| CEMT_28 | 64 | F | CLL |
| CEMT_29 | 43 | M | CLL |
| CEMT_30 | 39 | F | CLL |
| CEMT_4 | 66 | M | CLL |
| CEMT_5 | 50 | F | SLL |
| CEMT_6 | 52 | M | CLL |
| CEMT_92 | 62 | M | CLL |
| CEMT_93 | 66 | M | CLL |
| CEMT_94 | 66 | M | CLL |
| CEMT_95 | 61 | M | CLL |
| CEMT_96 | 91 | M | CLL |
| CEMT_97 | 52 | M | CLL |

**Table S2: Number of Ig-reads aligned to each chromosome/contig of GRCh38.** The pair-end 75bp reads aligned to the Ig-transcripts were considered as Ig-reads. Average number of Ig-reads mapped in 16 CEMT CLL samples are showed here; chromosomes with >=100 mapped reads are listed here. Columns are reverse sorted by the average number of reads mapped.

| Chromosome/contig | maximum | mean | percent |
| --- | --- | --- | --- |
| chr14 | 5339200 | **2595824.94** | 99.030 |
| chr15 | 66113 | **9474.94** | 0.361 |
| chr16_KI270728v1_random | 58871 | **4268.94** | 0.163 |
| chr14_KI270726v1_random | 53897 | **4223** | 0.161 |
| chr16 | 20527 | **4155.94** | 0.159 |
| chr11 | 4583 | 450.19 | 0.017 |
| chr2 | 1276 | 371.44 | 0.014 |
| chr1 | 879 | 307.13 | 0.012 |
| chr3 | 1504 | 263.69 | 0.010 |
| chr5 | 1328 | 244.06 | 0.009 |
| chr8 | 1419 | 225.69 | 0.009 |
| chr6 | 827 | 217.38 | 0.008 |
| chr19 | 650 | 207 | 0.008 |
| chr12 | 623 | 182.44 | 0.007 |
| chr17 | 482 | 174.5 | 0.007 |
| chr4 | 627 | 155.25 | 0.006 |
| chr7 | 495 | 152.31 | 0.006 |
| chrM | 417 | 145.81 | 0.006 |
| chr9 | 265 | 108.5 | 0.004 |
| chrX | 323 | 101.44 | 0.004 |

**Table S3: *IGHV* sequences identified by assembly of whole transcriptome and assembly from putative Ig loci in GSE66228 dataset**(Blachly *et al.*, 2015)**.** The difference is highlighted in bold font.

| RNA-seq ID | *IGHV* from whole transcriptome | % *IGHV* mutation | *IGHV* from putative Ig loci | % *IGHV* mutation |
| --- | --- | --- | --- | --- |
| SRR1814049 | IGHV1-69*04 | 0.3 | IGHV1-69*04 | 0.3 |
| SRR1814050 | IGHV1-18*04 | 0 | IGHV1-18*04 | 0 |
| SRR1814064 | IGHV3-11*01 | 2 | IGHV3-11*01 | 2 |
| SRR1814054 | IGHV3-74*01 | 5.4 | IGHV3-74*01 | 5.4 |
| SRR1814049 | IGHV4-59*02 | 8.5 | IGHV4-59*02 | 8.5 |
| SRR1814058 | IGHV3-66*02 | **1.4** | IGHV3-66*02 | **0.7** |
| SRR1814056 | IGHV4-34*01 | 0 | IGHV4-34*01 | 0 |
| SRR1814057 | IGHV2-70*01 | 0.3 | IGHV2-70*01 | 0.3 |
| SRR1814063 | IGHV3-74*03 | 8.8 | IGHV3-74*03 | 8.8 |
| SRR1814065 | IGHV3-53*01 | 6.1 | IGHV3-53*01 | 6.1 |
| SRR1814053 | IGHV2-70*01 | 0.3 | IGHV2-70*01 | 0.3 |
| SRR1814052 | IGHV1-46*01 | 0 | IGHV1-46*01 | 0 |
| SRR1814061 | IGHV1-3*01 | 0 | IGHV1-3*01 | 0 |
| SRR1814055 | IGHV3-21*01 | 0 | IGHV3-21*01 | 0 |
| SRR1814059 | IGHV3-48*03 | 2.4 | IGHV3-48*03 | 2.4 |
| SRR1814062 | IGHV1-46*01 | 0 | IGHV1-46*01 | 0 |

**Table S4:** ***IGHV* mutation status in CEMT CLL samples by CRIS.** CRIS reconstructed *IGHV-D-J* segments of Ig-transcripts. NA is used in cases where *IGHD* genes were absent in *IGH* sequence.

| CEMT_ID | IGHV | % mutation | IGHD | IGHJ | # transcript | # clonotype |
| --- | --- | --- | --- | --- | --- | --- |
| CEMT_1 | IGHV4-59*01 | 10.3 | IGHD3-22*01 | IGHJ6*02 | 13 | 6 |
| CEMT_4 | IGHV4-31*03 | 0 | IGHD3-3*01 | IGHJ6*03 | 16 | 4 |
| CEMT_5 | IGHV3-21*01 | 2.4 | NA | IGHJ6*02 | 19 | 10 |
| CEMT_6 | IGHV3-23*01 | 15.6 | IGHD3-10*01 | IGHJ4*02 | 7 | 5 |
| CEMT_25 | IGHV1-3*01 | 11.5 | IGHD6-13*01 | IGHJ4*02 | 15 | 6 |
| CEMT_26 | IGHV4-34*01 | 3.1 | IGHD3-22*01 | IGHJ4*02 | 13 | 5 |
| CEMT_27 | IGHV3-30-3*01 | 0 | IGHD2-8*01 | IGHJ4*02 | 24 | 13 |
| CEMT_28 | IGHV3-7*01 | 5.8 | IGHD4-17*01 | IGHJ4*02 | 18 | 4 |
| CEMT_29 | IGHV1-8*01 | 0.3 | NA | IGHJ3*02 | 8 | 2 |
| CEMT_30 | IGHV3-23*01 | 0.3 | IGHD2-2*02 | IGHJ6*03 | 7 | 4 |
| CEMT_92 | IGHV3-11*01 | 0 | IGHD3-10*01 | IGHJ5*02 | 19 | 10 |
| CEMT_93 | IGHV3-7*01 | 4.8 | IGHD3-22*01 | IGHJ2*01 | 29 | 17 |
| CEMT_94 | IGHV3-48*01 | 3.3 | IGHD6-13*01 | IGHJ4*02 | 6 | 4 |
| CEMT_95 | IGHV3-53*01 | 1.7 | IGHD1-1*01 | IGHJ4*02 | 15 | 7 |
| CEMT_96 | IGHV4-34*01 | 5.8 | IGHD3-10*01 | IGHJ4*02 | 12 | 6 |
| CEMT_97 | IGHV3-23*01 | 5.8 | IGHD3-10*01 | IGHJ6*03 | 11 | 6 |

**REFERENCES**

Altschul,S.F. *et al.* (1990) Basic local alignment search tool. *J. Mol. Biol.*, **215**, 403–410.

Beekman,R. *et al.* (2018) The reference epigenome and regulatory chromatin landscape of chronic lymphocytic leukemia. *Nat. Med.*, **24**, 868–880.

Blachly,J.S. *et al.* (2015) Immunoglobulin transcript sequence and somatic hypermutation computation from unselected RNA-seq reads in chronic lymphocytic leukemia. *Proc. Natl. Acad. Sci. U. S. A.*, **112**, 4322–4327.

Bolger,A.M. *et al.* (2014) Trimmomatic: a flexible trimmer for Illumina sequence data. **30**, 2114–2120.

Grabherr,M.G. *et al.* (2011) Full-length transcriptome assembly from RNA-Seq data without a reference genome. *Nat. Biotechnol.*, **29**, 644–652.

Li,H. and Durbin,R. (2009) Fast and accurate short read alignment with Burrows-Wheeler transform. *Bioinformatics*, **25**, 1754–60.

Quinlan,A.R. and Hall,I.M. (2010) BEDTools: a flexible suite of utilities for comparing genomic features. **26**, 841–842.

Ronique Giudicelli,V. *et al.* IMGT/GENE-DB: a comprehensive database for human and mouse immunoglobulin and T cell receptor genes.
